# Molecular Hydrogen Confers Resistance to Rice Stripe Virus

**DOI:** 10.1128/spectrum.04417-22

**Published:** 2023-02-22

**Authors:** Yudong Shao, Feng Lin, Yueqiao Wang, Pengfei Cheng, Wang Lou, Zhaoyun Wang, Zhiyang Liu, Dongyue Chen, Wei Guo, Ying Lan, Linlin Du, Yijun Zhou, Tong Zhou, Wenbiao Shen

**Affiliations:** a Jiangsu Key Laboratory for Food Quality and Safety-State Key Laboratory Cultivation Base, Institute of Plant Protection, Jiangsu Academy of Agricultural Sciences, Nanjing, Jiangsu Province, China; b College of Life Sciences, Laboratory Center of Life Sciences, Nanjing Agricultural University, Nanjing, China; Institute of Biotechnology

**Keywords:** *Arabidopsis*, disease resistance, molecular hydrogen, rice, rice stripe virus, salicylic acid

## Abstract

Although molecular hydrogen (H_2_) has potential therapeutic effects in animals, whether or how this gas functions in plant disease resistance has not yet been elucidated. Here, after rice stripe virus (RSV) infection, H_2_ production was pronouncedly stimulated in Zhendao 88, a resistant rice variety, compared to that in a susceptible variety (Wuyujing No.3). External H_2_ supply remarkably reduced the disease symptoms and RSV coat protein (CP) levels, especially in Wuyujing No.3. The above responses were abolished by the pharmacological inhibition of H_2_ production. The transgenic *Arabidopsis* plants overexpressing a hydrogenase gene from Chlamydomonas reinhardtii also improved plant resistance. In the presence of H_2_, the transcription levels of salicylic acid (SA) synthetic genes were stimulated, and the activity of SA glucosyltransferases was suppressed, thus facilitating SA accumulation. Genetic evidence revealed that two SA synthetic mutants of *Arabidopsis* (*sid2-2* and *pad4*) were more susceptible to RSV than the wild type (WT). The treatments with H_2_ failed to improve the resistance to RSV in two SA synthetic mutants. The above results indicated that H_2_ enhances rice resistance to RSV infection possibly through the SA-dependent pathway. This study might open a new window for applying the H_2_-based approach to improve plant disease resistance.

**IMPORTANCE** Although molecular hydrogen has potential therapeutic effects in animals, whether or how this gas functions in plant disease resistance has not yet been elucidated. RSV was considered the most devastating plant virus in rice, since it could cause severe losses in field production. This disease was thus selected as a classical model to explore the interrelationship between molecular hydrogen and plant pathogen resistance. In this study, we discovered that both exogenous and endogenous H_2_ could enhance plant resistance against *Rice stripe virus* infection by regulating salicylic acid signaling. Compared with some frequently used agrochemicals, H_2_ is almost nontoxic. We hope that the findings presented here will serve as an opportunity for the scientific community to push hydrogen-based agriculture forward.

## INTRODUCTION

Rice stripe virus (RSV, genus *Tenuivirus*) has been considered the most devastating plant virus in rice, since it could cause severe rice stripe disease and even give rise to enormous losses in field production, especially in East Asia (1). Other plants of the family *Poaceae* (2), and even other species such as Arabidopsis thaliana, were also observed to be infected by RSV (3). The spread of this virus from plant to plant was mainly mediated by the small brown planthopper (SBPH, *Laodelphax striatellus* Fallen) in a circulative-propagative manner (4). RSV-infected plants commonly exhibit alternated yellow and chlorotic stripes, curly and drooping leaves, premature wilting, and other adverse phenotypes (5, 6). It has been reported that the host immune responses appeared in the early stage of symptom development, in which the defense-response-associated processes were activated (7).

In plants, salicylic acid (SA) regulates both local and systemic acquired disease resistance (8). During pathogen infection, most of the free SA is converted to SA-2-*O*-*β*-d-glucoside (SAG) and then transported to the vacuole for degradation (9). When challenged with RSV, the resistant allele of rice *STV11* was found that encodes a sulfotransferase to catalyze the conversion of SA into sulfonated SA (1). Besides, the hypersensitive-induced reaction gene family contributes to plant basal resistance against RSV via an SA-dependent pathway (10).

Hydrogen gas (H_2_) is known as the structurally simplest of all gases in nature. Many microalgae and cyanobacteria can express hydrogenases that reduce protons to gaseous H_2_ (11). Since the antioxidant property of H_2_ in animals was observed by Ohsawa et al. (12), H_2_ is gradually regarded as a therapeutic medical gas (13). In plants, H_2_ has been found to act as one of the beneficial gasotransmitters in responses to various abiotic stresses (14), including cadmium exposure in alfalfa (15) and cucumber (16), and salinity in *Arabidopsis* (17).

It has been reported that hypoxia was a driving factor for enhancing H_2_ production in green microalga (18). In addition, several potential stimulus sources, including abscisic acid (ABA), jasmonic acid (JA), salinity, and drought stress, for H_2_ production have been described (19). Further results discovered that ABA elicited a rapid and sustained H_2_ production in *Arabidopsis*, causing a reduction in the stomatal aperture and enhancing drought tolerance (20). H_2_ control of rice aluminum tolerance was closely associated with the reconstruction of gibberellin acid (GA)/ABA balance and gene expression modulated by miRNA (21). Exogenous H_2_ was also closely associated with lateral root formation (22) and adventitious rooting (23). However, the role of endogenous H_2_ in plant responses to pathogen infection has not yet been elucidated.

In this study, we aimed to investigate whether H_2_ might be the key factor required for a strong hypersensitive RSV-resistance response. Since the specific synthesis pathway(s) of H_2_ in plants are still unclear (24), most of the experiments on exploring H_2_ functioning in plants were based on pharmacological approaches (hydrogen-rich water or solid-state hydrogen storage material) (25). However, the exogenous application of H_2_ may not fully mimic the endogenous H_2_ functioning in plant physiology. The transgenic *Arabidopsis* plants where the hydrogenase1 gene (*CrHYD1*) from Chlamydomonas reinhardtii was overexpressed (26), were also used to provide the genetic evidence to explore the functions of endogenous H_2_. For the above reasons, pharmacological, molecular biological, and genetic methods were adopted and combined to reveal the mechanisms underlying H_2_-regulated rice resistance to RSV. This study might open a new window for applying H_2_ to improve plant disease resistance.

## RESULTS

### A possible link between endogenous H_2_ production and RSV resistance in rice.

**FIG 1:**
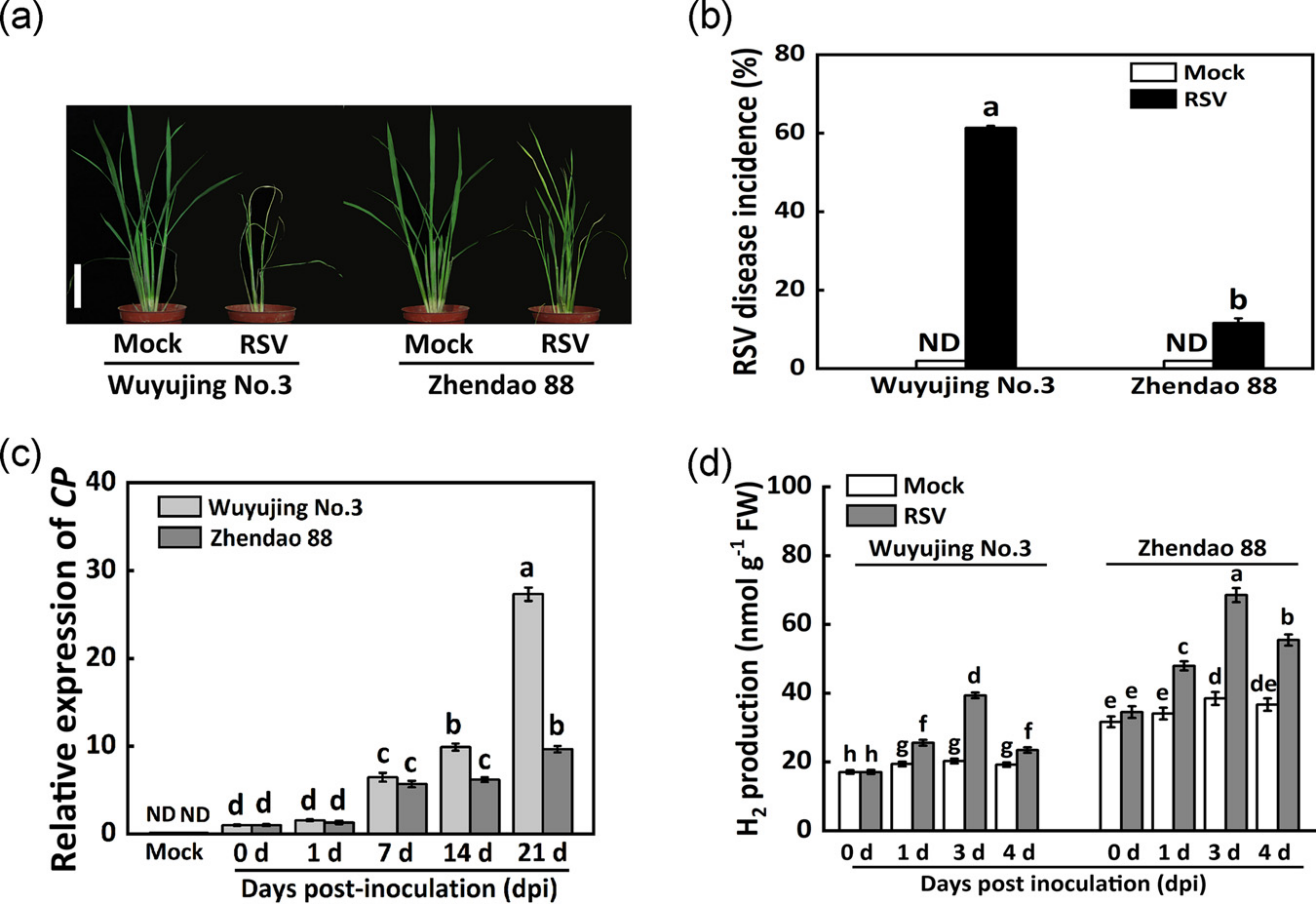
The differences between Wuyujing No.3 and Zhendao 88 after RSV inoculation. (a) Morphological comparison at 30 days postinfection (dpi) when the symptoms were observed. Scale bar, 5 cm. (b) RSV disease incidence (%) calculated with the data of 30 dpi. Mock: plants were inoculated with noninfected SBPH. Thirty lines of each variety with three replicates were analyzed. (c) Expression levels of RSV CP transcript in the two varieties. (d) Endogenous H_2_ production determined at different time points. Experimental data were from three independent biological replicates. ND, not detected.

After RSV challenge, the different phenotypes between the RSV-susceptible rice cultivar (Wuyujing No.3) and RSV-resistance rice variety (Zhendao 88) were assessed under our experimental conditions. As shown in Fig. 1a, the more severe symptoms caused by RSV inoculation were observed in Wuyujing No.3 compared to Zhendao 88. The results from RSV disease incidence revealed that approximately 60% of the Wuyujing No.3 plants were infected with RSV, while the percentage of symptomatic rice plants was just about 10% in Zhendao 88 (Fig. 1b). To test virus accumulation, the transcriptional levels of RSV coat protein (CP) in the above two rice varieties were further measured. Similar to the phenotypes, the qPCR results displayed that the accumulations of RSV RNA in Wuyujing No.3 were higher than that in Zhendao 88 at 14 and 21 days postinfection (dpi) (especially; Fig. 1c).

By using gas chromatography (GC), we further observed that endogenous H_2_ production was gradually stimulated after RSV inoculation in both Wuyujing No.3 and Zhendao 88 (in particular), peaking at 3 dpi (Fig. 1d). Additionally, basal levels of endogenous H_2_ in Zhendao 88 were higher than that in RSV-susceptible rice cultivar Wuyujing No.3 under the mock condition. The above results clearly indicated that endogenous H_2_ might be related to the different responses to RSV infection in two rice cultivars.

### Rice resistance to RSV infection is enhanced by exogenous H_2_ application.

To examine the role of H_2_ in rice defense against RSV, hydrogen-rich water (HRW) was used as a H_2_ donor to treat rice seedlings, at the same time period of RSV inoculation.

**FIG 2:**
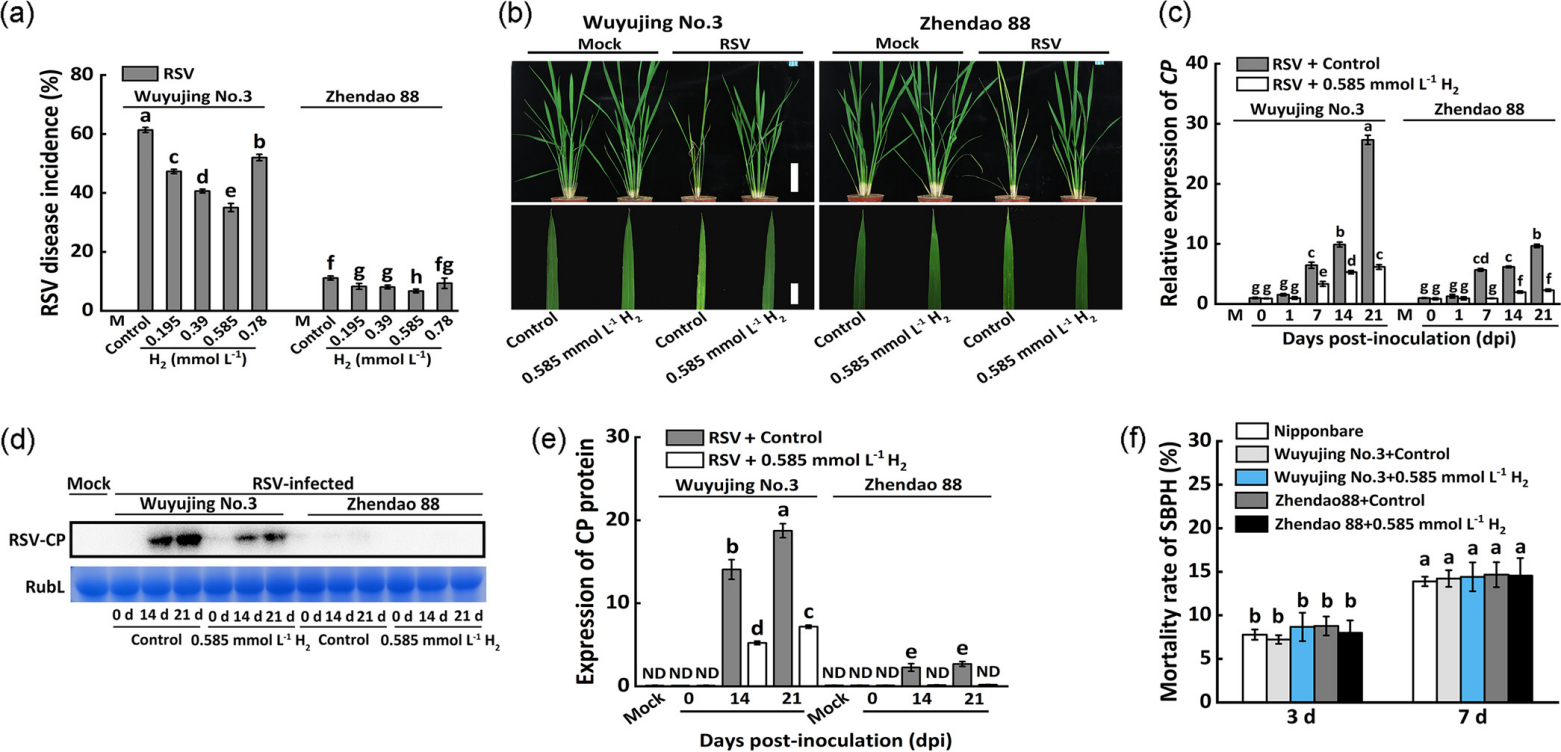
Exogenous H_2_ treatment enhances rice resistance to RSV. (a) RSV disease incidence (%) in response to different concentration of exogenous H_2_ at 30 dpi. (b) Morphological comparison between Wuyujing No.3 and Zhendao 88 at 30 dpi under exogenous H_2_ supply. Scale bar: 5 cm. (c) Expression level of RSV CP. (d) Accumulation of RSV CP determined through Western blot assay using an RSV CP-specific antibody under H_2_ treatment at 0, 14, and 21 dpi, and (e) quantified by ImageJ software. (f) Mortality rate of SBPH at 3 and 7 dpi. Rice cv. Nipponbare was used as SBPH susceptible control. ND, not detected.

Based on the pilot experiments (Fig. 2a), culture solution containing 0.585 mmol L^−1^ H_2_ showed the best effects, especially in the susceptible variety Wuyujing No.3, in which the RSV disease incidence declined from 62.5% to 38.3% at 30 dpi. The HRW containing 0.585 mmol L^−1^ H_2_ was thus selected for further analysis. After inoculation, disease symptoms were induced by RSV infection both in Wuyujing No.3 (in particular) and Zhendao 88 compared to the mock (Fig. 2b). After being treated with exogenous H_2_, however, both of the two varieties above displayed fewer yellow and green stripes than water-treated plants (control).

Meanwhile, the increased expression levels of *CP* in both rice varieties caused by RSV inoculation (7, 14, and 21 dpi) were significantly reduced by exogenous H_2_ treatment (Fig. 2c). Especially, it remained at a lower level in Zhendao 88, compared to Wuyujing No.3, the RSV-susceptible rice cultivar. Compared to the controls, the lower level of RSV-CP protein was also detected in exogenous H_2_-treated Wuyujing No.3 plants (14 and 21 dpi; Fig. 2d, e), and the RSV-CP was not obviously detected in Zhendao 88 throughout the process of RSV infection with or without H_2_ treatment. The above results clearly supported the conclusion that the rice resistance to RSV infection was obviously enhanced by exogenous H_2_ application.

The evaluation of mortality rate of SBPHs showed that Wuyujing No.3 and Zhendao 88 had a similar sensitivity to SBPHs regardless of exogenous H_2_ addition (Fig. 2f), indicating that H_2_-mediated RSV resistance is independent of SBPHs resistance.

### Inhibition of H_2_ production makes rice more susceptible to RSV.

**FIG 3:**
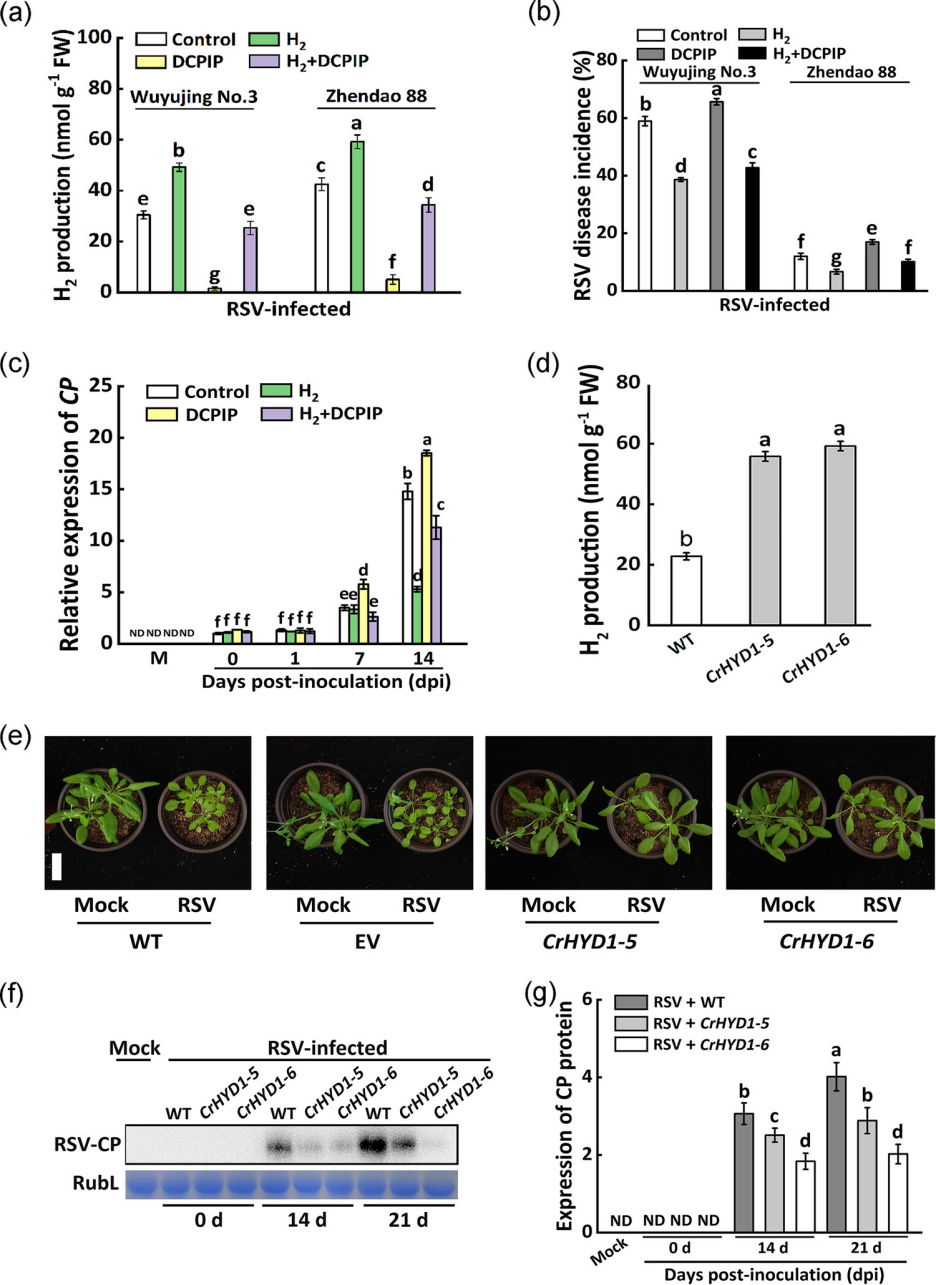
H_2_ positively regulates plant resistance to RSV. (a) H_2_ production in response to exogenous H_2_ or DCPIP, alone or its combination. Control: water treatment after RSV inoculation at 30 dpi. (b) RSV disease incidence (%). (c) Accumulation of RSV CP in Wuyujing No.3 analyzed through RT-qPCR under different treatments at 0, 1, 7, and 14 dpi. (d) H_2_ production in WT and *CrHYD1* transgenic *Arabidopsis* seedlings. (e) Symptoms of WT and *CrHYD1* transgenic *Arabidopsis* seedlings observed at 18 dpi. Scale bar: 1 cm. (f) Accumulation of RSV CP in WT and *CrHYD1* transgenic *Arabidopsis* seedlings at 0, 14, and 21 dpi, and (g) quantified by ImageJ software. ND, not detected.

To further assess the role of endogenous H_2_ in response to RSV infection, the rice seedlings were cotreated with RSV inoculation and 50 μmol L^−1^ 2,6-dichlorophenolindophenol (DCPIP), a putative inhibitor of H_2_ synthesis. It was obviously observed that compared to the control samples, H_2_ production *in vivo* was remarkably inhibited by DCPIP in both Wuyujing No.3 and Zhendao 88 plants, and the increased endogenous H_2_ content achieved by the addition of exogenous H_2_ was also sensitive to the DCPIP addition (Fig. 3a), reflecting the complexity of H_2_ synthesis in plants.

Consistently, compared to the controls, the disease incidence of Wuyujing No.3 and Zhendao88 was significantly increased when DCPIP was applied alone, and the cotreatment of exogenous H_2_ plus DCPIP abolished the DCPIP-induced increase in RSV disease incidence of rice (Fig. 3b). Although the alternations in transcriptional levels of CP were not affected by H_2_ and/or DCPIP application at 0 and 1 dpi, the similar trends (corresponding to RSV disease incidences; Fig. 3b) of CP expression were observed in Wuyujing No.3 at 7 and 14 dpi (especially; Fig. 3c). These results further confirmed that the endogenous H_2_ played a key role in rice resistance to RSV infection.

### Genetic evidence for endogenous H_2_ is required for plant resistance to RSV.

Since the biosynthetic pathway(s) of H_2_ has not yet been elucidated in higher plants and *Arabidopsis* could be infected by RSV (3), the transgenic *Arabidopsis* plants overexpressed *CrHYD1* (Gene ID in NCBI: 5718949; a hydrogenase gene from Chlamydomonas reinhardtii) (26) was used to reveal the endogenous H_2_ functioning in plant RSV resistance. Similar to previous studies (26, 27), the basal levels of endogenous H_2_ in *CrHYD1-5* and *CrHYD1-6* transgenic lines were significantly higher than that in WT plants (Fig. 3d). Compared with WT and control *Arabidopsis* transformed with empty vector (EV), the milder virus symptoms were observed in two *CrHYD1* lines at 18 dpi (Fig. 3e).

Subsequent Western blot analyze showed that RSV CP protein accumulation in WT plants displayed increasing trends after RSV inoculation (Fig. 3f, g). Comparatively, the increased CP protein levels caused by RSV inoculation were obviously reduced in two *CrHYD1* lines at 14 and 21 dpi. The genetic evidence above clearly indicated that endogenous H_2_ might be required for plant resistance to RSV.

### SA signaling pathway is involved in H_2_-induced RSV resistance.

**FIG 4:**
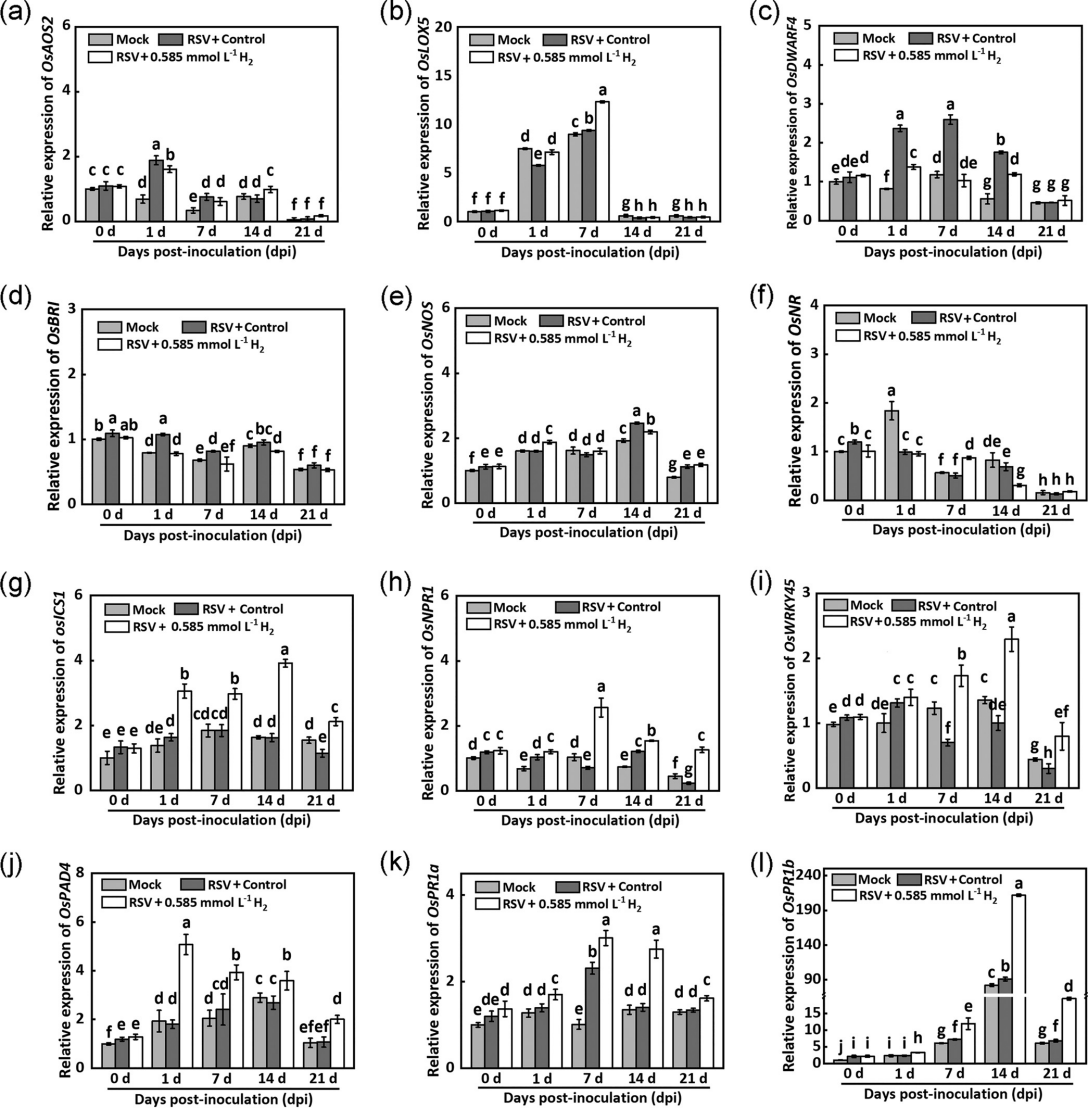
Reprogramming of the transcript levels of jasmonic acid (JA) pathway-related genes *OsAOS2* (a) and *OsLOX5* (b), brassinosteroid related-genes *OsDWARF4* (c) and *OsBRI1* (d), nitric oxide-related genes *OsNOS* (e) and *OsNR1* (f), salicylic acid (SA) biosynthetic and signaling genes *OsICS1* (g), *OsNPR1* (h), *OsWRKY45* (i), *OsPAD4* (j), *OsPR1a* (k), and *OsPR1b* (l) in Wuyujing No.3 upon exogenous H_2_ supply.

Previous reports have proved that some phytohormones and gasotransmitters were involved in the H_2_-regulated plant responses to a series of stresses (14, 25). Accordingly, the transcriptional profiles of some representative JA biosynthesis pathway-related genes (*OsAOS2*, Gene ID in NCBI: 4332121; *OsLOX5*, Gene ID in NCBI: 4333823), brassinosteroid related-genes (*OsDWARF4*, Gene ID in NCBI: 4332134; *OsBRI1*, Gene ID in NCBI: 4324691), nitric oxide-related genes (*OsNOS*, Gene ID in NCBI: 4328006; *OsNR1*, Gene ID in NCBI: 4345795), and SA-related genes (*OsICS1*, Gene ID in NCBI: 9268489; *OsNPR1*, Gene ID in NCBI: 4327315; *OsWRKY45*, Gene ID in NCBI: 4338413; *OsPAD4*, Gene ID in GenBank: CX118864.1; *OsPR1a*, Gene ID in NCBI: 4342317; and *OsPR1b*, Gene ID in GenBank: EF061247.1) in Wuyujing No.3 were then analyzed by RT-qPCR. Compared with the RSV treatment, the expression levels of the above genes were affected to various degrees by RSV plus 0.585 mM H_2_ treatment (Fig. 4). Among them, the changes in the expression of the six SA-related genes were markedly observed, showing great increases in RSV plus H_2_ treatment compared to RSV alone at 1, 7, 14, and 21 dpi. The above results indicated that SA signaling pathway might play a key role in H_2_-enhanced RSV resistance.

### SA might act downstream of H_2_ signaling in RSV resistance.

**FIG 5:**
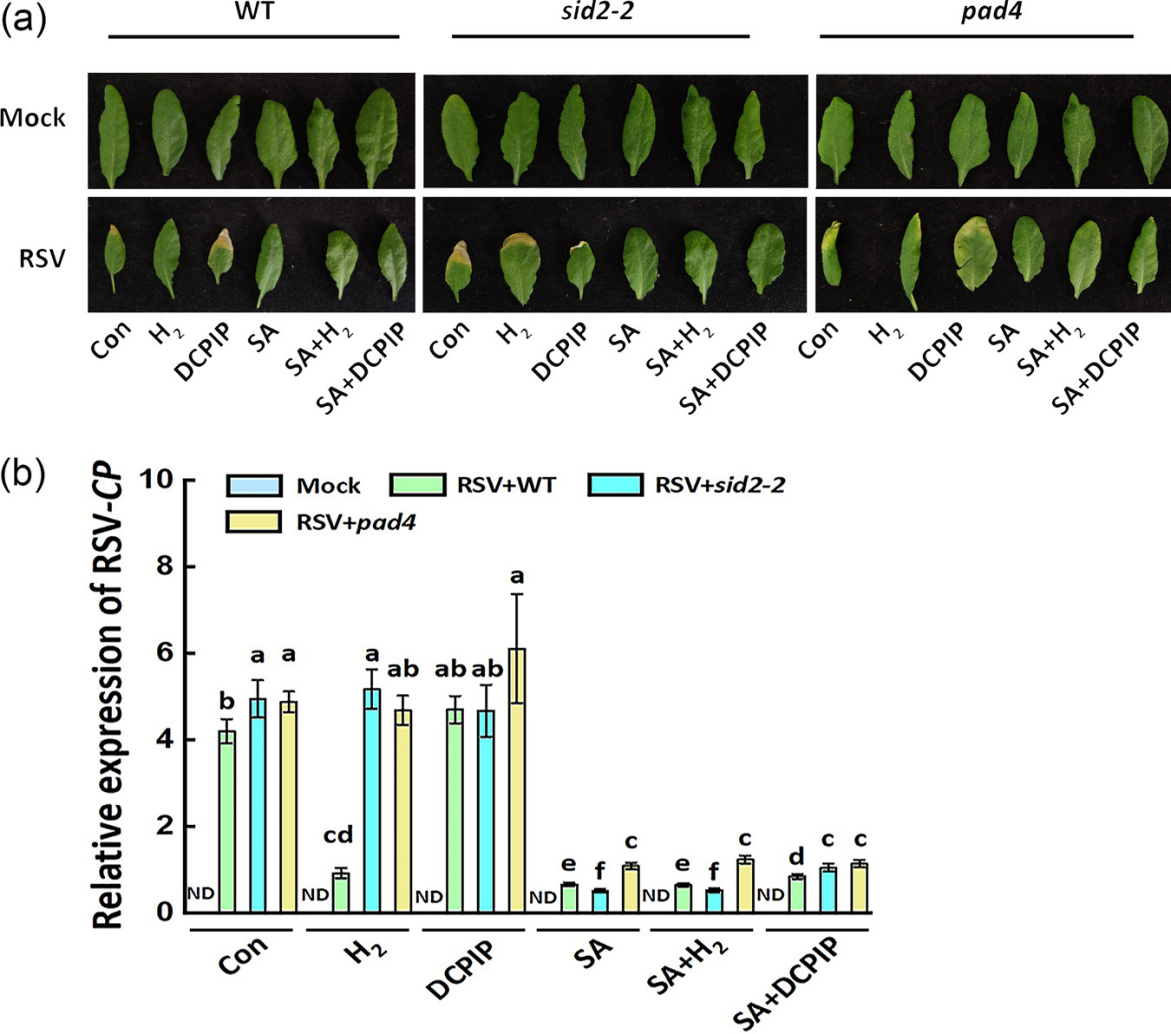
Arabidopsis thaliana SA synthetic mutants showing impaired plant resistance against RSV achieved by H_2_. (a) Symptoms and (b) relative expression level of RSV CP of *Arabidopsis sid2-2* and *pad4* mutant seedlings observed at 18 dpi under H_2_, DCPIP, SA, SA+H_2_, and SA+DCPIP treatments. Scale bar, 1 cm. ND, not detected.

Subsequently, SA induction deficient (*sid2-2*) mutant (28) and phytoalexin deficient 4 (*pad4*) mutant (PAD4 is a regulator of defense responses and acts upstream from SA; Zhou et al. [29]) were used to explore the SA functioning in the enhanced plant RSV resistance achieved by endogenous H_2_. In the chemical-free control condition (Con), the increased susceptibility to RSV was clearly observed in two mutants at 18 dpi (Fig. 5a), which was obviously abolished by SA addition, showing the lower susceptibility to RSV in both WT and two mutants. The results above do confirm that SA homeostasis was closely associated with RSV resistance.

With the administration with H_2_ or DCPIP, the contrasting response in RSV resistance was observed in WT plants. Compared to control treatment, for example, the RSV resistance was increased by H_2_ application, but reduced in the presence of DCPIP, further emphasizing the importance of H_2_ homeostasis. For the two SA-deficient mutants, unlike the responses in WT, no such obvious alternation was observed after H_2_ or DCPIP addition. We also noticed that no additive effects of H_2_ and SA were observed in both WT and mutants. And the SA control of RSV resistance was slightly suppressed by DCPIP addition in WT and *sid2-2* mutants.

Subsequent experiment revealed that the changes in the relative expression of RSV-CP displayed an approximately negative correlation with the resistance against RSV under the identical experimental conditions (Fig. 5b). Together, these results suggested that SA might act downstream of H_2_ signaling in RSV resistance.

### H_2_-induced RSV resistance is associated with free-SA accumulation.

**FIG 6:**
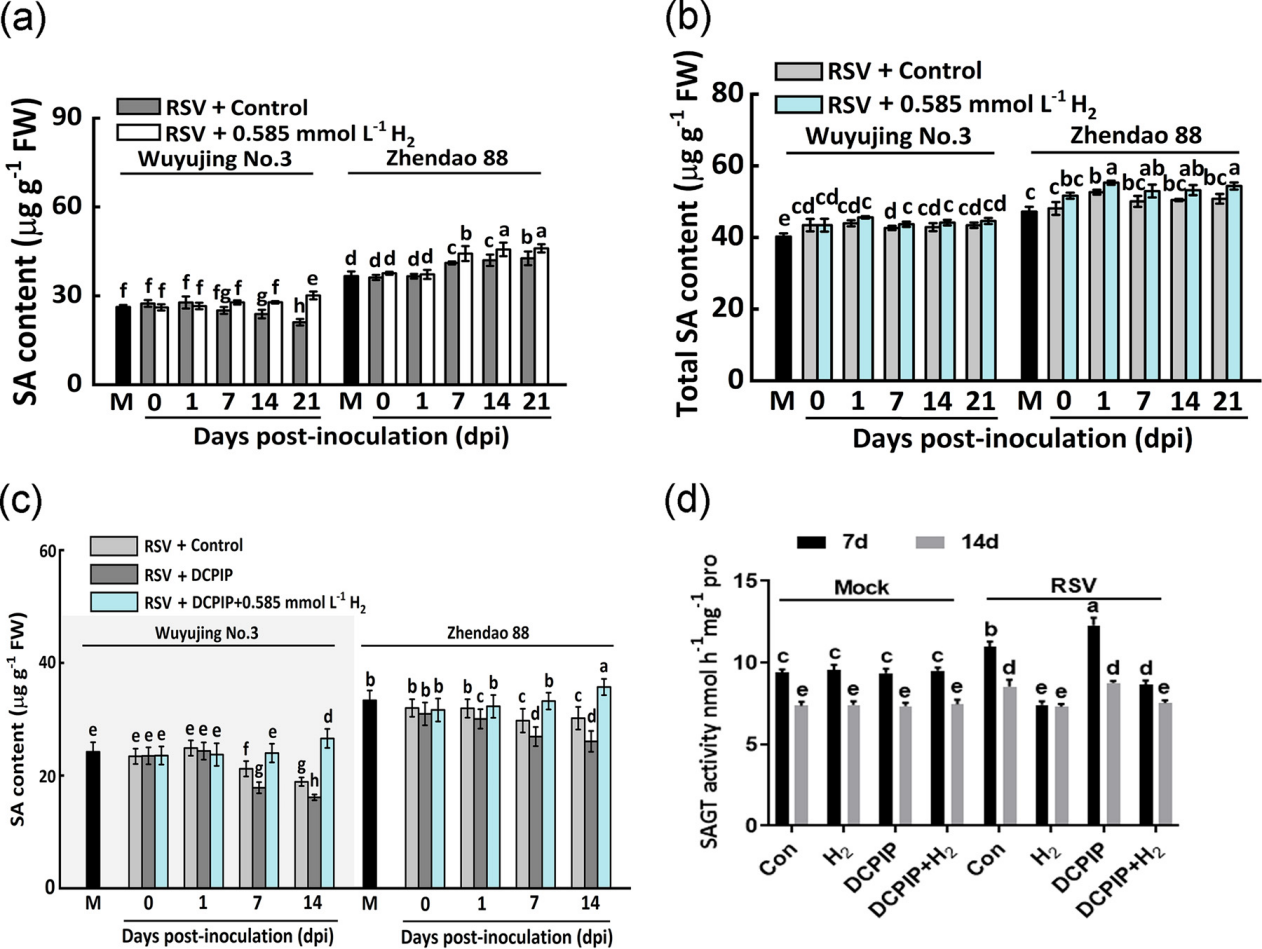
H_2_ regulates free SA content by affecting SAGT activity in rice. (a) Free SA and (b) total SA contents in response to H_2_ treatment at 0, 1, 7, 14, and 21 dpi. (c) Free SA content and (d) SAGT activity in response to H_2_ or DCPIP, alone or their combination in Wuyujing No.3. M represents mock group.

In order to further confirm the relationship between H_2_ and SA in RSV resistance, the accumulation of SA was analyzed. After RSV inoculation, free SA content in the resistant variety Zhendao88 was facilitated, and remained at higher levels compared with the susceptible variety Wuyujing No.3 (Fig. 6a). Meanwhile, the decreasing tendency of free SA was observed in Wuyujing No.3. In the presence of H_2_ addition, the SA contents in two cultivars were significantly increased as early as 7 dpi. A weaker or not-significant change of total SA contents was observed during the whole stage of RSV infection under the identical conditions (Fig. 6b).

Compared with the RSV plus control group, the free SA content in Zhendao 88 was remarkably reduced when DCPIP was applied (7 and 14 dpi; Fig. 6c). The above negative effects caused by DCPIC were markedly reversed in the presence of exogenous H_2_ (RSV+DCPIP+H_2_). Combined with phenotypes (Fig. 3b, 5), the above results suggested that H_2_ might influence the conversion between free SA and bound SA, thus enhancing rice resistance to RSV infection.

A previous study showed that SA glucosyltransferases (SAGT) could catalyze the conversion of free SA to SAG (30). To confirm the above speculation, the SAGT activity was measured in the susceptible variety Wuyujing No.3. As expected, the activity of SAGT was almost identical in the mock group (four treatments: Con, H_2_, DCPIP, and DCPIP plus H_2_) at 7 and 14 dpi (Fig. 6d). After RSV inoculation, SAGT activity in the control group was significantly induced at both 7 and 14 dpi, compared with those in the mock group. As expected, exogenous H_2_ reduced SAGT activity at 7 dpi and remained at the same level at 14 dpi. A contrasting response was observed in the presence of DCPIP at 7 dpi. While at 14 dpi, the difference between control and DCPIP groups was not observed. Interestingly, compared to DCPIP alone, the increased SAGT activity was abolished when H_2_ was added together at 7 and 14 dpi. Combined with the changes in free and total SA content, the above results indicated that H_2_-induced RSV resistance is closely associated with free-SA accumulation.

## DISCUSSION

Since it acts as a selective antioxidant (12), molecular hydrogen was shown to have therapeutic and/or preventive effects on various reactive oxygen/nitrogen species-related inflammatory diseases, such as ischemia/reperfusion injury, radiation-induced heart disease, and so on (31). As reactive oxygen species (ROS) also play important roles in plant responses under abiotic and biotic stresses, the function of H_2_ in regulating ROS homeostasis has been proved (24). More recently, H_2_ has been considered a potential gasotransmitter in plants (14), since it is closely associated with plant growth and development, and abiotic stress response (25, 32). However, although the protective effect of H_2_ in postharvest tomato fruit resistance to Botrytis cinerea has been discovered (33), its functioning in enabling growing plants to cope with biotic stress is still elusive.

Here, we first discovered that when challenged with RSV, the endogenous H_2_ production was stimulated strongly in rice (Fig. 1d). More importantly, the RSV-induced endogenous H_2_ in Zhendao 88 (an RSV-resistance rice variety) kept higher levels than those in Wuyujing No.3, an RSV-susceptible rice cultivar (Fig. 1). This is a new finding, although the detailed synthetic pathway(s) for H_2_ in plants is still unclear (14, 34). Subsequent genetic and pharmacological evidence confirmed that H_2_, when applied exogenously or endogenously, could confer resistance to RSV, especially in RSV-susceptible rice cultivar (Fig. 2, 3), but failed in two Arabidopsis SA synthetic mutants (Fig. 5). The participation of SA was further presented (Fig. 4 to 6). Therefore, our data provide a new molecular basis for the differential resistance of plants to RSV.

Besides, the requirement for H_2_ in plant resistance to RSV has some other evidence. First, the disease incidence of Wuyujing No.3 and particularly in Zhendao88 was significantly intensified when DCPIP, a putative inhibitor of H_2_ synthesis in plants (26, 35), was applied together (Fig. 3a to c). Importantly, although we admitted that the possibility that DCPIP influences other enzyme activities (and therefore might lead to significant impact to rice immunity) is not easily ruled out, DCPIP addition in our experiments did inhibit endogenous H_2_ production (Fig. 3a). Second, the hypersensitivity to RSV in Wuyujing No.3 was significantly abolished after exogenously applied with HRW (Fig. 2), a normally used H_2_ supply method in plants (36). Finally, genetic evidence revealed the positive effects of overexpression of the hydrogenase gene (*CrHYD1*) from Chlamydomonas reinhardtii on both endogenous H_2_ production and resistant phenotypic against RSV in *Arabidopsis* (Fig. 3d to g).

We also noticed that the comparatively strong resistance to RSV was observed in *CrHYD1-6* compared to the *CrHYD1-5* line, which might be related to the relatively higher endogenous H_2_ level in *CrHYD1-6* plants. In rice, the basal levels of endogenous H_2_ and SA in Zhendao 88 (an RSV-resistance rice variety) were higher than those in Wuyujing No.3 (an RSV-susceptible rice cultivar) without RSV inoculation (Fig. 1d and 6a). These findings in basal levels of H_2_ and SA might partially explain discrepancies upon RSV attack in the above two cultivars and help to identify the causes of different phenotypes. Therefore, besides SA metabolism, the identification of potential gene(s) responsible for endogenous H_2_ production will be the next priority for hydrogen biology.

Further BLAST analysis of CrHYD1 protein sequence in rice showed the cytosolic Fe-S cluster assembly factor Nuclear Architecture Related 1 (NAR1) annotated as putative hydrogenase in rice in NCBI, with 28.31% identity with CrHYD1 in Chlamydomonas reinhardtii. However, when the rice NAR1 protein sequence was executed for a BLAST search in Chlamydomonas reinhardtii, the uncharacterized protein CHLRE_03g200550v5 (37.69%) instead of CrHydA1 (29.37%) has the highest identity, suggesting that the NAR1 might exist independently of HydA1 in Chlamydomonas reinhardtii. The similar result was obtained after the NAR1 protein in another model organism, Arabidopsis thaliana, executed by BLAST. Importantly, a previous study found that Fe hydrogenase-like proteins cannot metabolize hydrogen in some organisms (37). Collectively, we still have little information on the functions of the putative hydrogenase in rice in terms of hydrogen metabolism upon pathogen attack. These need further study.

Ample evidence confirmed that H_2_ may improve plant tolerance to abiotic stress via modulating hormone signaling pathways, including abscisic acid, ethylene, jasmonate acid, and so forth (19). H_2_ production is positively associated with auxin-induced lateral root formation via a nitrate reductase-dependent nitric oxide synthesis (22) and enhancement of aluminum tolerance in rice by altering the ratio of GA and ABA (21). In fact, several phytohormones have been reported for their close association with plant resistance against RSV, such as brassinosteroids and JA (38, 39). In this report, by detecting the expression level of key genes of several hormone signaling pathways (Fig. 4), we proposed that SA signaling transduction might be the possible candidate participating in the above positive functions of molecular hydrogen in both rice and Arabidopsis when challenging with RSV.

Previous reports have demonstrated that SA plays a central role in plant disease resistance (40) and participates in plant defense against virus (1, 41). In maize, resistance to sugarcane mosaic virus infection was most likely through the regulation of SA accumulation (42), which was involved in turnip mosaic virus resistance in Nicotiana benthamiana (43). The resistant allele of *STV11* conferring durable resistance to RSV encodes a sulfotransferase catalyzing the conversion of SA into sulfonated SA (1). In our subsequent experiments, two SA synthetic mutants (*sid2-2* and *pad4*) were analyzed for their response to RSV infection in the presence of H_2_. Both mutants have a defect in the accumulation of SA (28, 29). Although the recovery of RSV resistance in the WT was observed in the presence of exogenous application of H_2_, no such phenomenon was discovered in the above two SA signaling mutants (Fig. 5), indicating that SA might act as the downstream signaling component of molecular hydrogen signaling against biotic stress. Interestingly, the SA control of RSV resistance was slightly suppressed by DCPIP addition in WT and *sid2-2* mutants, suggesting that a complex signaling process between H_2_ response and some specific SA synthesis metabolism pathway(s) might exist, which need to be illustrated more clearly in the future.

The expression levels of several genes related to SA signaling pathway were analyzed upon H_2_ treatment after RSV inoculation (Fig. 4g to l). *PAD4* encodes a possible lipase (44) and functions upstream from salicylic acid signaling (29). The major SA biosynthesis gene *isochorismate synthase 1* (*ICS1*) is required for SA accumulation (45). The transcriptional coactivator NPR1 (nonexpressor of pathogenesis-related genes 1) is a master regulator of local and systemic resistance associated with SA pathway (46). Additionally, Rice WRKY45 transcription factor plays a crucial role in disease resistance mediated by the SA signaling pathway (47). Our molecular results showed that six SA-related genes (*OsICS1*, *OsNPR1*, *OsWRKY45*, *OsPAD4*, *OsPR1a*, and *OsPR1b*) might be involved in molecular hydrogen control of plant disease resistance against RSV (Fig. 4). Furthermore, exogenous H_2_ treatment suppressed SAGT activity and therefore induced SA accumulation, which could be used to explain the inhibited viral accumulation in the whole stage of RSV infection (Fig. 6).

**FIG 7:**
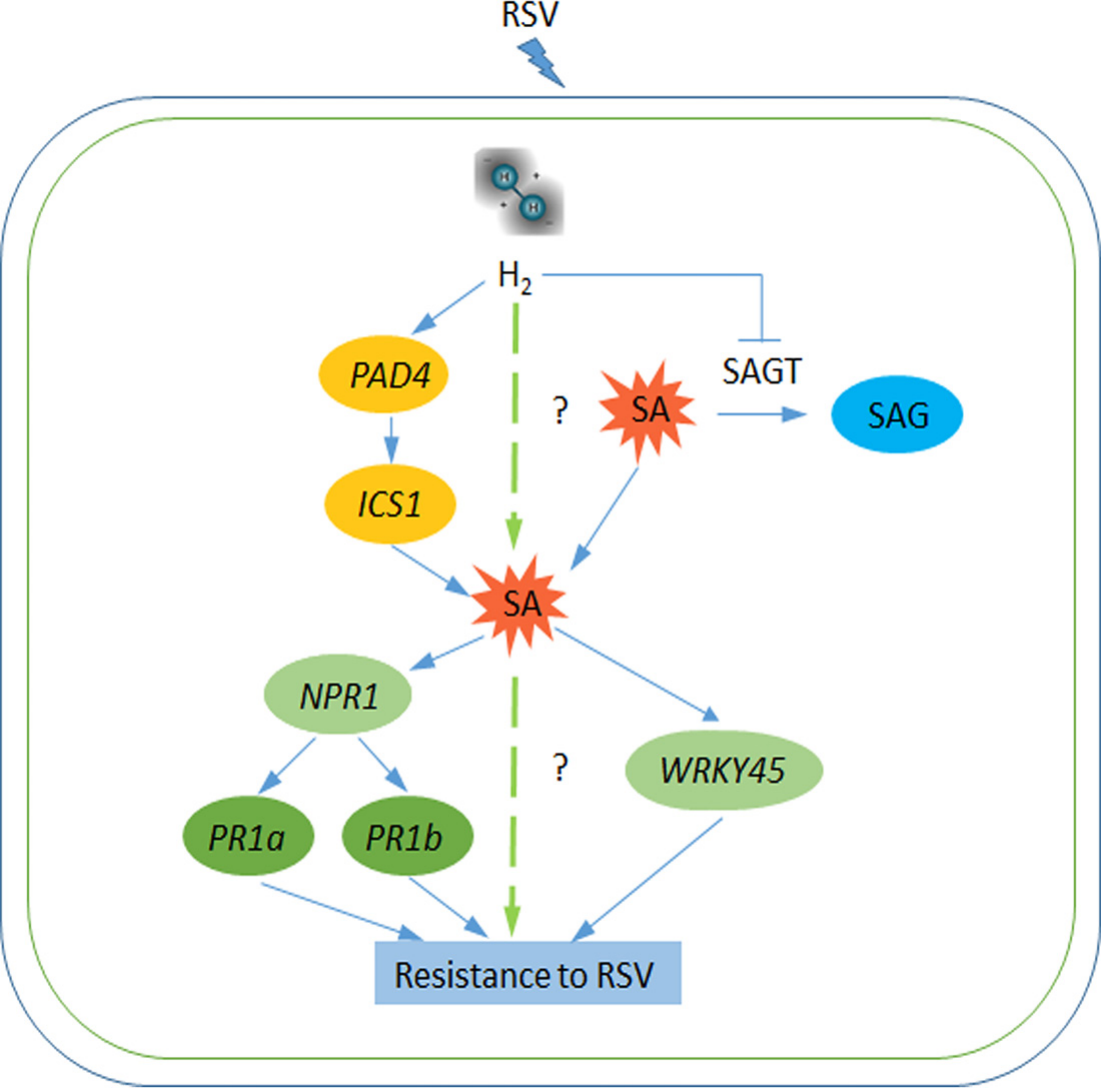
A model describing H_2_ conferring resistance to RSV. Molecular hydrogen could trigger SA signaling by reprogramming some genes (*PAD4* and *ICS1*) related to SA biosynthesis, and decreasing the SAGT activity to reduce the conversion from free SA to SAG. The increased SA triggers a series of SA signaling genes (*NPR1*, *PR1a*, *PR1b*, and *WRKY45*), thus enhancing plant resistance against RSV infection.

Taken together, we proposed and summarized the following model (Fig. 7). When challenged with RSV, H_2_ can be stimulated in plants, and this gas not only induces the expression level of *PAD4* and *ICS1*, acting as an upstream SA signaling pathway, but also suppresses SAGT activity and thus restrains the conversion from free SA to SAG. Finally, SA accumulation and its targeted genes (*NPR1*, *PR1a*, *PR1b*, and *WRKY45*) might be involved in H_2_ control of enhancing plant antiviral defense against RSV.

In fact, earlier studies proved that some chemical reagents, including melatonin, nitric oxide, and SA, could enhance disease resistance against plant pathogens attack (4). However, with the requirement of low-carbon society, the excessive application of agrochemicals is gradually occupying the antithesis of green agriculture (48). Compared with some frequently used agrochemicals, H_2_ is an almost nontoxic gasotransmitter. This study might open a new window for expanding the applied scope of hydrogen-based agriculture. We also hope that the findings presented here will serve as an opportunity for the scientific community to push the hydrogen agriculture forward.

## MATERIALS AND METHODS

### Plant materials and growth conditions.

Rice (Oryza sativa L.) seeds of Wuyujing No.3 (a susceptible cultivar to RSV [49]) and Zhendao 88 (a resistance cultivar to RSV [49]) were used in this study. The Arabidopsis thaliana ecotype Columbia-0 (Col-0; WT), SA induction deficient (*sid2-2*) mutant (28), phytoalexin deficient 4 (*pad4*) mutant (50), two lines of transgenic *Arabidopsis* with the overexpressed *hydrogenase 1* (Gene ID in NCBI: 5718949) from Chlamydomonas reinhardtii (*CrHYD1-5* and *CrHYD1-6*), and corresponding empty vector (EV) plants (26) were used in this study.

After surface sterilization, rice seeds were germinated in distilled water under dark surroundings at 27°C for about 2 days. Then the seedlings were transferred to a greenhouse with 14/10 h (26°C/21°C) light/dark cycle at 160 μmol m^−2^ s^−1^ irradiation.

Seeds of Arabidopsis thaliana were vernalized in darkness at 4°C for 3 days to vernalization, and then sown on 1/2 Murashige-Skoog medium and cultured for 7 days. The Arabidopsis thaliana seedlings were then transferred to flowerpots (peat: vermiculite, 1:3) and cultured in a greenhouse with 16/8 h (23°C/20°C) light/dark cycle at 120 μmol m^−2^ s^−1^ irradiation.

### Hydrogen-rich water (HRW) preparation.

A hydrogen gas generator (SHC-300, Saikesaisi Hydrogen Energy Co., Ltd., China)-generated purified H_2_ (99.99%, vol/vol) was bubbled into 1,000 mL distilled water at a rate of 170 mL min^−1^ for 30 min to create saturated HRW, in which the concentration of dissolved H_2_ was 0.78 mM. The fresh saturated HRW was immediately diluted to 75% (0.585 mM), 50% (0.39 mM), and 25% saturability (0.195 mM).

### Artificial inoculation of RSV.

RSV-infected rice plants were harvested from the field in Jiangsu Province, China. SBPH nymphs were reared on the RSV-positive rice plants for 3 days to acquire the virus and maintained on Wuyujing No. 3 rice seedlings. Viruliferous SBPHs were determined by using dot-ELISA (3).

For rice, at least 30 2.5-leaf-stage seedlings per cultivar were randomly selected, and each seedling was inoculated with two viruliferous (RSV) or virus-free (mock) SBPHs for 2 days as described before (49, 51). For *Arabidopsis*, 30 4-leaf-stage plants were inoculated with 10 viruliferous (RSV) or virus-free (mock) SBPHs per plant for 4 days as described by Sun et al. (3). Three replicates were performed for each accession. Subsequently, all insects in plants were removed, and seedlings were cultured in the greenhouse. Seedling tissues were sampled from the inoculated plants at various days postinoculation (dpi).

### Chemicals and treatments.

All chemicals were obtained from Sigma-Aldrich (St. Louis, MO, USA), except those with statements. Kimura Solution B solution was used for rice treatment prepared as described by Wang et al., (52), and 2.5-leaf-stage rice plants (Wuyujing No.3 and Zhendao 88) were incubated in 500-mL Kimura solution B solutions containing 0.195, 0.39, 0.585, and 0.78 mmol L^−1^ H_2_, respectively. Fresh nutrient solutions were replaced every 3 days during the experiment. *Arabidopsis* was watered directly by HRW containing 0.585 mmol L^−1^ H_2_.

We selected 50 μmol L^−1^ 2,6-dichlorophenolindophenol (DCPIP; a putative inhibitor of H_2_ synthesis) (35) and 500 μmol L^−1^ SA for treatment. When inoculated by transferring viruliferous SBPHs, 30 rice or *Arabidopsis* plants per replicate were treated with 0.585 mmol L^−1^ H_2_ or 50 μmol L^−1^ DCPIP, 500 μmol L^−1^ SA alone, or a different combination at the same time.

### Determination of H_2_ content.

Endogenous H_2_ production was determined by gas chromatography (GC; Tianmei GC7900, Tianmei Scientific Instrument Co., Ltd., China), based on the previous method (36).

### SA measurement.

The concentration of SA was measured according to a previous method (53). After being ground in liquid nitrogen, each sample (0.2g) was homogenized in 1 mL 90% methanol, and the organic phase was evaporated *in vacuo* to dryness.

For the free SA samples, the residues were dissolved in 800 μL 5% trichloroacetic acid and an equal volume of mixed solution containing ethyl acetate, cyclohexane, and isopropyl alcohol (50:50:1, vol/vol/vol). For the total SA samples, the residues were dissolved in 800 μL sodium acetate buffer (0.1 M), and the pH was adjusted to 1.0 by hydrochloric acid. After heating at 80°C for 30 min, the released free SA was reextracted as the free SA samples.

After dryness, the organic phase was dissolved in 500 μL 40% methanol. Filtered with 0.22 μm organic filter membrane, the samples were analyzed by ultraperformance liquid chromatography (UPLC; Agilent 1260, Agilent, Palo Alto, USA).

### Quantitative real-time PCR analysis.

Total RNA was extracted from seedlings (0.1 g) with TRIzol reagent (Invitrogen, MD, USA), and cDNA was synthesized using the PrimeScript RT reagent kit with a gDNA Eraser (TaKaRa, Dalian, China). qPCR was carried out with SYBR qPCR Master Mix kit (TaKaRa, Dalian, China). Specific qPCR primers are listed in Table S1 in the supplemental material. Relative transcript levels were converted to a linear form with the formula Q = 2^−ΔΔCt^ (54).

### Western blot analysis.

Seedling tissues were extracted using protein extraction buffer (Beyotime, USA) with protease inhibitor (Beyotime; wt/vol = 1:100). Total 20 μg sample protein was separated on 10% sodium dodecyl sulfate-polyacrylamide gel electrophoresis gels through electrophoresis and transferred onto polyvinylidene fluoride membranes. The primary antibody was diluted (1:4,000) in blocking buffer for 1.5 h, then the secondary antibody was added (1:8,000) and incubated for 2h. Antibody binding was detected by ECL (enhanced chemiluminescence) (Pierce, USA). Anti-RSV-CP antibody used for diagnosis of RSV-positive plant seedlings was provided by Jianxiang Wu, Zhejiang University (55).

### Measurement of SA glucosyltransferase activity.

SA glucosyltransferase activity was determined based on previous method (9, 30) with some modifications. Assay mixtures contained sample proteins with a final concentration of 50 mmol L^−1^ sodium phosphate buffer (pH 7.6), 100 μmol L^−1^ SA, and 0.5 mmol L^−1^ UDP glucose. The reaction mixture was incubated at 30°C for 60 min and stopped by addition of 0.3 mL trichloroacetic acid. Products were analyzed by high-pressure liquid chromatography (HPLC; Agilent 1260, Agilent, Palo Alto, USA).

### Statistical analysis.

Each experiment was conducted in triplicate, and data were presented as the mean with standard deviations (SD). Statistical analyses were carried out with SPSS 23.0 (Armonk, USA). Differences were analyzed using one-way analysis of variance (ANOVA), and the error bars represent the SDs of different treatments with three biological replicates. Different letters indicate a significant difference (with *P* value < 0.05). All analyses were conducted with Origin 9.0 software.

### Data availability.

The data sets and materials supporting the findings of this article are available from the corresponding author upon reasonable request.
